# Protein *O*-Fucosyltransferase 1 Undergoes Interdomain Flexibility in Solution

**DOI:** 10.3390/molecules26082105

**Published:** 2021-04-07

**Authors:** Erandi Lira-Navarrete, María Carmen Pallarés, Fabio Castello, Maria J. Ruedas-Rama, Angel Orte, Anabel Lostao, Ramón Hurtado-Guerrero

**Affiliations:** 1 Institute of Biocomputation and Physics of Complex Systems (BIFI), University of Zaragoza, 50018 Zaragoza, Spain; erandi@bifi.es; 2 Instituto de Nanociencia y Materiales de Aragón (INMA), CSIC-Universidad de Zaragoza, 50009 Zaragoza, Spain; maricarmenpallaresmatute@gmail.com; 3 Laboratorio de Microscopías Avanzadas (LMA), Universidad de Zaragoza, 50018 Zaragoza, Spain; 4 Departamento de Fisicoquímica, Facultad de Farmacia, Universidad de Granada, 18071 Granada, Spain; e-fabiocastello@ugr.es (F.C.); mjruedas@ugr.es (M.J.R.-R.); 5 Fundación ARAID, 50018 Zaragoza, Spain; 6 Copenhagen Center for Glycomics, Department of Cellular and Molecular Medicine, School of Dentistry, University of Copenhagen, 2200 Copenhagen, Denmark

**Keywords:** glycosyltransferases, *O*-fucosylation, protein dynamics, atomic force microscopy, single-molecule methods

## Abstract

Protein *O*-fucosyltransferase 1 (PoFUT1) is a GT-B fold enzyme that fucosylates proteins containing EGF-like repeats. GT-B glycosyltransferases have shown a remarkable grade of plasticity adopting closed and open conformations as a way of tuning their catalytic cycle, a feature that has not been observed for PoFUT1. Here, we analyzed *Caenorhabditis elegans* PoFUT1 (*Ce*PoFUT1) conformational behavior in solution by atomic force microscopy (AFM) and single-molecule fluorescence resonance energy transfer (SMF-FRET). Our results show that this enzyme is very flexible and adopts mainly compact conformations and to a lesser extend a highly dynamic population that oscillates between compact and highly extended conformations. Overall, our experiments illustrate the inherent complexity of *Ce*PoFUT1 dynamics, which might play a role during its catalytic cycle.

## 1. Introduction

PoFUT1 is a glycosyltransferase (GT) present in the endoplasmic reticulum that catalyzes the transfer of a fucose (Fuc) residue from GDP-Fuc directly into Ser/Thr residues of some proteins containing EGF-like repeats [1,2]. There are around l00 proteins that can be potentially modified by PoFUT1 [2,3], being the Notch receptors the most studied of them. These receptors are transmembrane proteins that are the core of the Notch signaling pathway. They can have ~29–36 EGF like-repeats [4], that can be fucosylated by PoFUT1 although not all have the fucosylation acceptor sites [5]. For *O*-fucosylation to take place two criteria must be fulfilled: (1) the EGF like-repeats must have the consensus sequence C_2_-X-X-X-X-S/T-C_3_ where X can stand for any amino acid [6]; and (2) the EGF like-repeats should be correctly folded [7]. The importance of the addition of a Fuc residue into Notch receptors has been deeply studied in the past years, e.g., a Fuc moiety promotes the interaction between Notch and its ligands [8,9]. This was revealed by the recent crystallographic data that demonstrated how a Fuc residue located at Notch1 EGF12 contributes significantly to the contact with delta like ligand 4 (DLL4) [8] and Jagged1 [9]. In addition, the complex between Notch1 and Jagged1 was further stabilized by another important interaction between a Fuc moiety at Notch1 EGF8 and N298 of Jagged1 EGF3 [9]. The Fuc moiety is further elongated with a *N-acetylglucosamine* (GlcNAc) residue by the Fringe GTs [4]. Fringe activity has an important tuning function in Notch signaling in *Drosophila melanogaster*. Particularly, it potentiates Notch binding to Delta while impedes Notch interaction with Serrate (counterpart for the mammalian Jagged) [10,11]. The scenario is rather more complex in higher eukaryotes [12,13]. A chaperone-like function has also attributed to PoFUT1 [14], though this has not been confirmed by additional experiments.

PoFUT1 is categorized as a GT-B inverting enzyme [15,16]. GTs can be classified as inverting or retaining and they can be further classified according to their folds into GT-A (containing one single Rossmann-like fold domain), GT-B (two separating Rossmann-like fold domains facing each other), and GT-C (membrane GTs without Rossmann-like fold domains) [17,18].GTs are enzymes with rather flexible and dynamic structures [19] that are mostly characterized by containing flexible loops that usually control and fine-tune the enzymatic reaction [20,21,22,23,24]. In the particular case of GT-B GTs, conformational changes are attributed mostly to the overall dynamic of both facing domains adopting the GT-B fold and specific regions of one of the domains. These are exemplified for some GTs namely glycogen synthase [25], GlcNAc transferase MshA [26] and mannosyltransferase PimA [27]. These enzymes have an open conformation in the enzyme-free form that shifts to a closed conformation upon sugar nucleotide binding. In PimA’s particular case, there is also a major conformational change involving a helical hairpin at the N-terminal domain which moves away from the rest of the protein in a conformation that may be beneficial for its interaction with membranes [27,28]. The recent structure of FUT8 complexed to GDP and an N-glycan derivative has also shown important conformational changes in which the open and closed states are dictated by a loop and the α-helix 10, which contribute to the catalytic cycle of this particular enzyme [29].

Several PoFUT1 crystal structures have been solved in free form and in the presence of GDP/GDP-fucose and different EGFs [14,15,29,30], evidencing that there are no major conformational changes and no interdomain dynamics. Folding of the EGFs as aforementioned is essential for enzyme recognition, and the groove-shaped EGF binding site located between the two PoFUT1 domains fits precisely the EGF shapes [31]. Here, we have identified significant dynamic structural changes in *Ce*PoFUT1 by performing different single-molecule techniques, holding molecular resolution. Combined atomic force microscopy (AFM) [32] and single-molecule fluorescence resonance energy transfer (SMF-FRET) [33] experiments have revealed the intrinsic complexity of this enzyme in solution.

## 2. Results

### 2.1. Plasticity of CePoFUT1 in Solution

**Figure 1:**
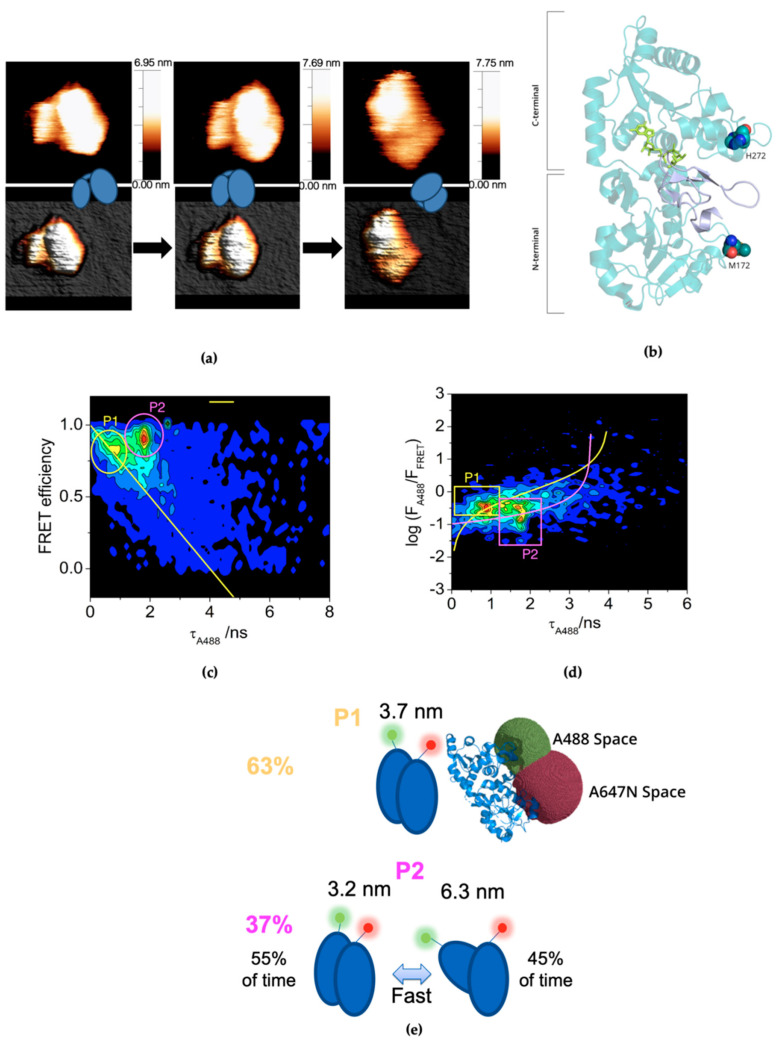
(**a**) Compact structures visualized in 2D and 3D for the same *Ce*PoFUT1 apo molecule in the presence of GDP in three consecutive collected AFM images. Images present a high-level contrast to show better the domains. The features appear next to outlined ball shapes that represent the species found. (**b**) Localization of residues M172 and H272 residues in *Ce*PoFUT1 structure. The figure depicts the structure of *Ce*PoFUT1 complexed with GDP-Fuc (PDB entry 3ZY4) and EGF1 (T101A). Note that the EGF1 shown in the structure comes from the crystal structure of mouse PoFUT1 (*Mm*PoFUT1) complexed to EGF1 (T101A) and GDP-Fuc (PDB entry 5KY3). *Ce*PoFUT1 and EGF1 are shown as a cartoon representation in teal and violet colors, respectively. GDP-Fuc is shown as green carbon atoms. The mutated residues are depicted as spheres. (**c** and **d**) Multiparametric SMF-FRET correlograms of dual-labeled *Ce*PoFUT1 in solution. Both correlograms of FRET efficiency vs. *τ*_A488_ (**c**) and log (*F*_A488_/*F*_FRET_) vs. *τ*_A488_ (**d**) show the presence of two *Ce*PoFUT1 populations. P1 (static population) and P2 (dynamic population) are shown in yellow and magenta, respectively. In both cases, the yellow line represents the theoretical relations between FRET efficiency and *τ*_A488_ for a static population. Magenta line in (**d**) represents a dynamic population interconverting between a low-FRET and a high-FRET state, according to Equation (2). (**e**) Percentages of P1 and P2 and estimated donor-acceptor distances for both populations.

To determine the dynamics of *Ce*PoFUT1 in solution at the single-molecule level, we performed both AFM and SMF-FRET experiments on *Ce*PoFUT1 in the free form. AFM images evidenced that this enzyme adopts mostly a monomeric form with different degrees of compactness. Sample immobilization for AFM imaging was weak, providing a high degree of freedom of movement, which on some occasions even made possible the observation of different opening degree for the same protein molecule by scanning the same area in different plane directions at higher rates as the example shown in Figure 1a. One of the observed conformations resembles the observed compact conformation in the crystallographic structures described previously [15,30,31], where the N- and C-terminal domains are facing each other exposing a clear cleft between them, where EGF repeats are located. Regarding the conformations found by AFM, most of them present heights between 6.6 nm (corresponding to the extended conformations) to 7.8 nm (exhibited by the more compact conformations). This is explained considering that in the more closed structures there is a certain overlap of the domains that causes the global height above the plane to slightly increase.

Since AFM is only capable of providing static information, as it shows snapshots of the different forms of *Ce*PoFUT1 in solution, we performed advanced, multiparametric SMF-FRET experiments to estimate the dynamics of this enzyme in solution. To conduct this type of experiment, we first made a double mutant of *Ce*PoFUT1 in which we mutated solvent-exposed residues, namely Met172 and His272 (Figure 1b), to Cys residues, and labeled them with a suitable FRET pair, namely the donor ATTO 488 (A488) and acceptor ATTO 647N (A647N) dyes (see Materials and Methods). The mutated residues were located in each domain of the enzyme and were far distant from both the substrates binding sites (Figure 1b). We thus studied the single-molecule FRET correlograms in terms of FRET efficiency (*E*), estimated through bursts intensity as *F*_FRET_/(*F*_FRET_ + γ·*F*_A488_), versus the donor fluorescence lifetime *τ*_A488_, as an orthogonal measure of the FRET efficiency, since *E* = 1 − *τ*_A488_/*τ*^0^_A488_. For A488, the fluorescence lifetime in the absence of acceptor, *τ*^0^_A488_, is 4.1 ns (https://www.atto-tec.com/). The *E* vs. *τ*_Donor_ in multiparametric SMF experiments is a powerful tool to unravel different subpopulations of individual molecules, even at very low count numbers [34]. Figure 1c shows a representative *E* vs. *τ*_A488_ correlogram for the dual-labeled *Ce*PoFUT1 in solution. Since *E* and *τ*_A488_ are directly related, all the single-molecule events arising from molecules in which the donor and the acceptor are at certain static distance should lay within a straight line, depicted in yellow in the correlogram. Strikingly, we clearly detected two different subpopulations of events in those correlograms: a first population, P1, that laid within the expected *E* vs. *τ*_A488_ straight line, and a second population, P2, whose peak clearly was out of the linear relation. Both subpopulations exhibited a high FRET efficiency, but P2 showed an anomalously high value for the donor fluorescence lifetime. Importantly, this feature in the *E* vs. *τ*_A488_ correlogram is indicative of a dynamic process altering the donor-acceptor distance, and hence the *E* and *τ*_A488_ values, occurring in a time frame faster than the diffusion time of the protein through the excitation volume (circa. 1 ms). For such cases of dynamics occurring on sub-millisecond time scales, the relation between *E* and *τ*_A488_ for individual bursts is no longer linear. For a more reliable analysis of such situations, it is preferred the use of *F*_A488_/*F*_FRET_ ratio (in logarithmic scale) versus *τ*_A488_ correlograms. A representative log(*F*_A488_/*F*_FRET_) vs. *τ*_A488_ correlogram from dual-labeled *Ce*PoFUT1 in solution is depicted in Figure 1d. In such representation of the single-molecule FRET data, a FRET pair population with a static distance would follow the yellow line in Figure 1d, according to the following equation [35]:

(1) $${\left(\frac{F_{A488}}{F_{\mathrm{FRET}}}\right)}_{\mathrm{static}}=\gamma \frac{\tau_{A488}}{\tau_{A488}^{0}-\tau_{A488}}$$

However, for a dynamic situation, with sub-millisecond kinetics, Equation (1) is no longer valid. Considering the simplest dynamics, that is a rapid fluctuation of the biomolecular structure between a high-FRET (compact) and a low-FRET (extended) state, the equation that relates the *F*_A488_/*F*_FRET_ ratio and *τ*_A488_ is given by [35]:

(2) $${\left(\frac{F_{A488}}{F_{\mathrm{FRET}}}\right)}_{\mathrm{dyn}}=\gamma \frac{\tau_{A488}^{\mathrm{low}\mathrm{FRET}}\cdot \tau_{A488}^{\mathrm{high}\mathrm{FRET}}}{\tau_{A488}^{0}\left[\tau_{A488}^{\mathrm{low}\mathrm{FRET}}+\tau_{A488}^{\mathrm{high}\mathrm{FRET}}-\tau_{A488}\right]-\tau_{A488}^{\mathrm{low}\mathrm{FRET}}\cdot \tau_{A488}^{\mathrm{high}\mathrm{FRET}}}$$

where $\tau_{A488}^{\mathrm{low}\mathrm{FRET}}$ and $\tau_{A488}^{\mathrm{high}\mathrm{FRET}}$ are the fluorescence lifetime values of the A488 donor in the extended, low-FRET and the compact, high-FRET states, respectively; whereas *τ*_A488_ is the experimental fluorescence lifetime value within the single-molecule burst.

In order to characterize the extended and compact conformations of the dynamic population of *Ce*PoFUT1, we globally fitted the single-molecule events in all the *F*_A488_/*F*_FRET_ vs. *τ*_A488_ correlograms, but only considering those events away from the static line. This fitting provided the values for $\tau_{A488}^{\mathrm{low}\mathrm{FRET}}$ and $\tau_{A488}^{\mathrm{high}\mathrm{FRET}}$ that best matched the obtained population, recovering the values of 3.5 and 0.4 ns, respectively, and giving rise to the dynamic population line depicted in magenta in Figure 1d. Furthermore, the relative position of the P2 subpopulation across this line provided information on the relative weight of the two conformations, having obtained that this dynamic population was 55% of the time in the compact conformation and 45% of the time in the extended conformation.

Our SMF-FRET results picture a complex scenario for *Ce*PoFUT1 in solution, revealing two types of populations that behaved differently. Population 1, P1, is a static population (at least static in the millisecond time regime) while population 2, P2, is a dynamic population, with fast dynamics (in the millisecond time regime), which oscillates between extended and compact conformations (Figure 1e). We also quantified the relative amount of each population by defining certain cutoff areas in the histogram and counting the events laying in each one of the regions (Figure 1d). Quantification of each population rendered 63 ± 3% of the protein in the static P1 and the 37 ± 3% in the dynamic P2.

In order to estimate donor-acceptor distances of the detected populations, we first calculated a Förster distance, *R*_0_, value of 4.7 nm for the FRET pair employed. We then obtained that in P1 the distance between the M172 and H272 positions is 3.7 nm, while in P2 distances vary from 3.2 nm (compact conformation) to 6.3 nm (extended conformation). The distance of 3.7 nm measured for the static P1 population is larger than the distance between the labeling positions, measured in the crystal structure of *Ce*PoFUT1 to be around 2.5 nm. Nevertheless, this difference between the measured distance and the labeling positions is usually found in single-molecule FRET experiments for high FRET efficiency values, such as the one found in this work. Apart from the flexibility of the dyes’ linkers, other sources of uncertainty are related to the environment of the dyes, the validity of the assumption of free rotation of the dyes, and uncertainty on the determination of the correction factors (see Materials and Methods) [36]. For a more robust comparison of the measured distance within the structure of the protein, we employed the computational method developed by Seidel and collaborators to assess donor-acceptor distance distributions and the expected estimated distance on a single-molecule FRET experiment [37]. This method computes the three-dimensional space occupied by the donor and the acceptor fluorophores, assess the inter-dye distance distribution, considering the flexibility of the dyes’ linkers, and estimates the expected FRET efficiency and measured distance, depending on the *R*_0_ value of the FRET pair. Using this method with the *Ce*PoFUT1 crystal structure and the A488 and A647N dyes attached through flexible linkers to the γ C of the H272 and M172 residues, respectively, we estimated the expected distance obtained through a FRET experiment to be 3.5 nm (Figure 1e, see Materials and Methods for details). Moreover, the flexibility of the linkers results in an inter-dye distance distribution of ±1.1 nm. These results are in excellent agreement with the inter-dye distance measured in our SMF-FRET experiments for the static P1 population.

Therefore, our experiments support that the static P1 population is closely related to the crystal structure of *Ce*PoFUT1, whereas the dynamic P2 form is a real different subpopulation, with the ability of reaching an opened conformation. Overall, this picture is consistent with the AFM data and reinforces the finding that *Ce*PoFUT1 is highly dynamic in solution with striking large conformational changes not identified previously on PoFUT1 crystallographic work or any other reported GT-B GTs.

### 2.2. Additional Plasticity in CePoFUT1 upon Binding Substrates and Products

**Figure 2:**
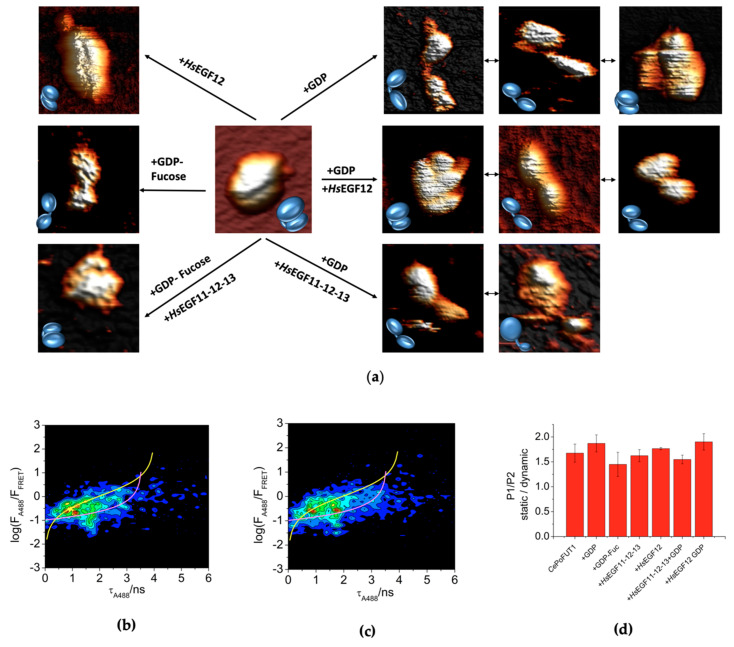
*Ce*PoFUT1 binding partners. (**a**) Representative AFM topography images taken in HEPES, pH 7.0 showing the main species found: highly-compact structure *Ce*PoFUT1 (center panel); transition from highly extended structure to compact structure in the presence of GDP (upper right panel); transition from compact structure to close extended in presence of GDP and *Hs*EGF12 (middle right panel); extended structure in presence of GDP and *Hs*EGF11-12-13 (bottom right panel); very compact structure in presence of GDP-Fuc and *Hs*EGF11-12-13 (bottom left panel); extended structure in presence of GDP-Fuc (middle left panel); and very compact structure in presence of *Hs*EGF12 (upper left panel). Images present a high-level contrast to show better the domains. The features appear next to outlined ball shapes that represent the species found. (**b** and **c**) Representative SMF-FRET correlograms (log(*F*_A488_/*F*_FRET_) vs. *τ*_A488_) of *Ce*PoFUT1 in the presence of GDP-Fuc (**b**) and GDP (**c**). (**d**) Ratio of the static and dynamic populations (P1/P2) of *Ce*PoFUT1 in the presence of different substrates/products.

To investigate whether the different conformations of *Ce*PoFUT1 had physiological relevance, we performed single-molecule experiments in the presence of substrate/products. AFM measurements displayed a more complex scenario to the visualized for the free form. While in the presence of GDP or GDP-Fuc, either in the presence or absence of the human EGF12 or EGF11-12-13, the enzyme displayed compact and extended conformations, in the presence of these repeats alone, only compact structures were observed, and in some cases very compact ones in which the cleft is almost not visible (Figure 2a). Note that the extended conformations are characterized by a large distance between both domains with no contact between them. This data suggest that extended structures were only visible either in the presence of GDP-Fuc or GDP, implying that these forms might be required as a previous state to binding to EGF repeats or as a strategy to trapping them. In addition, the data suggest that the closure of the EGF binding site takes place in the presence of an EGF repeat, and that the cleft is not present or poorly visualized likely due to the presence of a repeat on it. Note that the no observation of extended structures in the AFM measurements in the presence of EGFs only does not preclude that they do not exist in solution as demonstrated below.

SMF-FRET experiments showed that the distribution in percentages between populations did not significantly change in the presence of substrates/products and their combinations as shown in Figure 2b–d. The largest change was observed when GDP was added to the enzyme as visualized by AFM imaging (Figure 2a), however P1 was still the predominant population found for *Ce*PoFUT1.

### 2.3. Dimers of CePoFUT1

**Figure 3:**
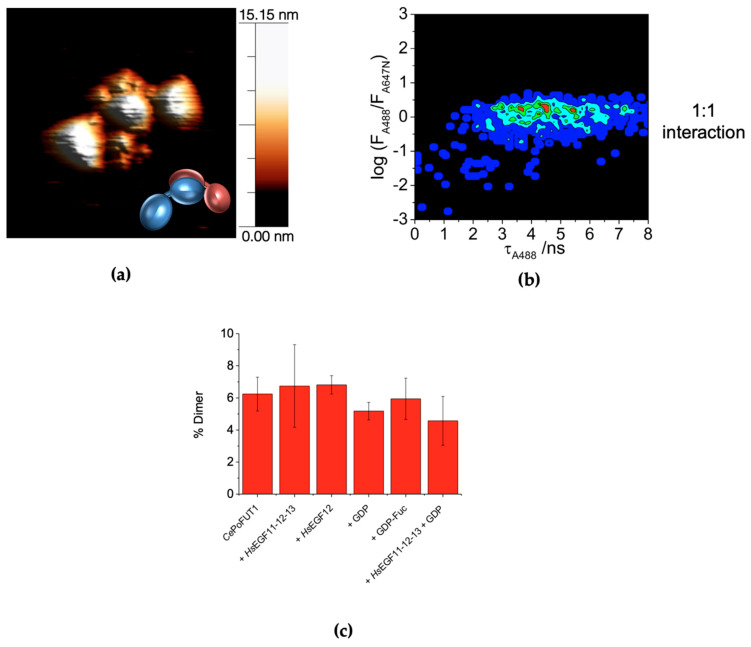
*Ce*PoFUT1 dimers. (**a**) AFM 3D image showing a *Ce*PoFUT1 dimer found when the enzyme was incubated with GDP and *Hs*EGF11-12-13. The feature appears next to outlined ball shapes that represent the species found. (**b**) Single-molecule coincidence correlogram (log(*F*_A488_/F_A647N_) vs. *τ*_A488_) of a mixture of singly-labeled *Ce*PoFUT1-A488 and *Ce*PoFUT1-A647N. (**c**) *Ce*PoFUT1 dimer percentage found in solution in the presence of different substrates.

In our AFM experiments, eventually, we were able to detect the presence of dimers of the enzyme though these were rarely present (Figure 3a). The existence of these dimers was more prevalent in the enzyme incubated with the acceptor substrate *Hs*EGF11-12-13. The dimers present a certain overlap of one of the domains that, together with the structural reorganization, makes the overall height above the plane twice that observed by the rest of the monomeric species (Figure 3a). We then studied this dimerization in solution at the single-molecule level using the singly-labeled constructs, *Ce*PoFUT1-A488 and *Ce*PoFUT1-A647N, mixed at stoichiometric ratio. The analysis of the SMF traces, specifically the directly excited A488 and A647N intensities, *F*_A488_ and *F*_A647N_, is capable of revealing dimerization through the identification of coincident single-molecule events. The quantification of the number of coincident events in both detection channels provides thermodynamic information on the amount and stability of complexes formed at the single-molecule level [38,39]. Importantly, this methodology does not require close proximity between the two fluorophores because they are independently excited, therefore, avoiding structural prerequisites for the experiments. Moreover, the PIE excitation scheme prevents any interference and spectral cross-talk between the detection channels, since excitation is achieved at two different time windows [40]. Using the SMF coincidence criterion we also observed a small percentage of *Ce*PoFUT1 dimers. The analysis of the single-molecule coincidence correlograms revealed a 1:1 stoichiometry interaction (Figure 3b). Quantification of dimeric *Ce*PoFUT1 revealed that only 4–6 % of the total protein was found in a dimeric state (Figure 3c) down at the single-molecule level, supporting the findings by AFM. Small variations on the dimers’ percentage was also observed when the protein was incubated with some of its substrates/products, having a slight increase in the presence of the acceptor *Hs*EGF12 (6.8 ± 0.6%) or *Hs*EGF11-12-13 (6.7 ± 2.6%), and decreasing with both GDP and *Hs*EGF11-12-13 (4.6 ± 1.2%) when compared with the free form (6.2 ± 1.1%) (Figure 3c).

Overall, our results suggest a highly dynamic behavior of *Ce*PoFUT1 in solution that can be fine-tuned by the presence of its substrates/products.

## 3. Discussion

GT-B GTs have been reported as flexible enzymes suffering conformational changes within compact forms. In particular, these GTs display the closure of the active site upon donor sugar nucleotide binding, which appears a prior step for enzyme catalysis [19]. However, this has never been observed for PoFUT1 from previous structural works [31], hence, it appears intuitive that EGF-like repeats by shielding the active site from the solvent would prevent potential conformational changes in PoFUT1 active site. Here and, to our surprise, by applying AFM and single molecule FRET methodologies, we demonstrate a highly flexible dynamic behavior for *Ce*PoFUT1 in solution. From our AFM data, we observed that *Ce*PoFUT1 displays different compact forms together with extended forms. The presence of these extended forms characterized by the long distance between both domains resemblance the extended forms visualized for GalNAc-Ts [22,41]. This family of GT-A folded GTs, possesses a unique C-terminal lectin domain joined to the N-terminal catalytic domain through a flexible linker, which gives these enzymes a great deal of flexibility to perform their catalytic activity in a specific manner [22,24,42].

Contrary to other GT-B GTs [25,26,27] and according to our AFM data, *Ce*PoFUT1 seems to prefer an extended conformation on the presence of either GDP or GDP-Fuc. This event could prepare the enzyme for EGF repeats recognition or as a strategy to trap EGF repeats. In this manner, a plausible hypothesis might consider that the trapping or binding to EGF repeats, prior binding to GDP-Fuc, might lead to the shift from an extended to a compact structure (see the different conformations visualized by AFM in the presence of GDP and *Hs*EGF12, Figure 2a), a transition which is required to perform catalysis optimally. Although the dynamics of this enzyme inferred by AFM is supported by single-molecule FRET, this latest technique exemplifies that a fixed percentage of different populations of *Ce*PoFUT1 is always present in solution and this does not vary in the presence of substrates/products. While the static population, which is characterized by the presence of compact structures, does not change, the dynamic population is likely to be subjected to different transitions between compact and extended conformations. Although *Ce*PoFUT1 is also found as a dimer in solution, this is a very rare form (around 6%), implying that they might not be relevant for catalysis.

Overall, we present strong evidences of a highly dynamic behavior for *Ce*PoFUT1, characterized by large conformational changes never seen before for any other GT-B GTs. For example, *Escherichia coli* glycogen synthase (*Ec*GS) shows an increase of 1.5 nm to transit from a compact to extended structure [25]. Here we report up to 3.1 nm increase during this transition for *Ce*PoFUT1, which is more than the double compared to the reported in *Ec*GS. However, and although this is a striking change, we do not know the exact role of this dynamic behavior. Interestingly, PoFUT2, a very close fucosyltransferase to PoFUT1 [3], does not have the same dynamic behavior shown by PoFUT1 [43]. Despite both enzymes performing similar reactions and they resemble at structural level (root-mean-square deviation of 3.03 Å on 174 Cα atoms), they diverge in many different aspects [16]. PoFUT2 presents a S_N_-2-like mechanism [43], which is the most common mechanism among inverting GTs [17,18,44], while PoFUT1 displays a S_N_-1-like mechanism [15,31] which is very atypical for an inverting GT. The recognition of their substrates also differs, while PoFUT2 recognizes disulfide bridges-containing thrombospondin repeats though mostly an intricated water molecules network [43], PoFUT1 deploys several amino acids in both N- and C- terminal domains for an optimal recognition [31]. Hence, it is tempting to speculate that the *Ce*PoFUT1 intrinsic dynamics in solution might have an important role during its catalytic cycle, which would require additional experiments for understanding of this particular behavior. Finally, we expect that our study will open a new perspective into the biophysical properties of PoFUT1 and its catalytic cycle.

## 4. Materials and Methods

### 4.1. Expression and Purification of CePoFUT1 Wild Type and Double Mutant M172C/H272C

*Ce*PoFUT1 was expressed and purified in *Pichia pastoris* X33 as previously reported [15]. Purification was simplified by passing *Ce*PoFUT1 supernatant through a HiTrap Blue column (GE Healthcare, Marlborough, MA, USA) and eluted in a gradient 0–1 M NaCl in 25 mM Tris-HCl pH 8.5. After this step, purified *Ce*PoFUT1 was loaded into a Superdex 75 XK26/60 column (GE Healthcare) previously equilibrated with 25 mM Tris-HCl pH 8.5, 150 mM NaCl. Protein was then dialyzed three times against 25 mM Tris-HCl pH 8.5. The single mutant M172C and the double mutant M172C/H272C were generated by GenScript (Leiden, The Netherlands) by performing site-directed mutagenesis. Purification was performed as described above.

### 4.2. Cloning, Expression and Purification of HsEGF12 and HsEGF11-12-13

The DNA sequences encoding residues 452-488 (*Hs*EGF12) and residues 412-526 (*Hs*EGF11-12-13) of human Notch1 were synthesized by GenScript (to be expressed in *E. coli*). The DNA sequences, containing at the 5′-end a recognition sequence for NcoI, at the 3′ end a stop codon, and a recognition sequence for BamHI, were cloned into a pUC57 vector (GenScript). Both constructs also contained a PreScission protease cleavage site. The two final constructs encoding for *Hs*EGF12 and *Hs*EGF11-12-13 were then subcloned into the protein expression vector pET32a (Novagen, Darmstadt, Germany), which also contains *Trx*. The resulting plasmids were transformed into Rosetta-gami 2 (DE3) cells.

Transformant cells were grown at 37 °C in 2xTY medium supplemented with 100 μg/mL ampicillin and 12 μg/mL tetracycline. Protein production was induced by adding 1 mM IPTG once cells reached an OD of 0.6 at 600 nm. Subsequently, they were harvested and disrupted by sonication 18 h after induction at 18 °C.

Solubilized *Hs*EGF12 or *Hs*EGF11-12-13 was loaded into a 1 × 5 mL HisTrap HP column (GE Healthcare) previously equilibrated with 25 mM Tris-HCl, 0.5 M NaCl, 20 mM imidazole and 1 mM CaCl_2_ pH 7.4. The proteins were eluted by applying an imidazole gradient from 20 mM to 500 mM. The eluted proteins were buffer exchanged (25 mM Tris-HCl pH 8, 150 mM NaCl and 1 mM CaCl_2_) using a HiPrep 26/10 Desalting Column (GE Healthcare). Then, the protein was incubated with PreScission protease (PP; GE Healthcare) at 4 °C for 16 h. Removal of Trx and PP was made by coupling a GST-Trap and a His Trap column (GE Healthcare). Non-bound protein was collected and further purified by size exclusion chromatography using a Superdex 75 XK26/60 column (GE Healthcare).

### 4.3. Labeling Reactions with Fluorescent Tags

The single-cysteine mutant M172C and the double mutant M172C/H272C of *Ce*PoFUT1 were labeled with fluorescent dyes for SMF studies. In detail, *Ce*PoFUT1-M172C mutant was labeled with either maleimide-modified ATTO488 (A488) or ATTO647N (A647N) dyes (ATTO-TEC GmbH, Siegen, Germany) in a 1:4 protein-to-dye ratio, to enhance dye reactivity. The reaction was performed in 25 mM Tris buffer, at pH = 8.5, shaking at room temperature (21 °C) for 12 h. The *Ce*PoFUT1-M172C/H272C mutant was sequentially and randomly labeled at positions 172 and 272 with the same dyes, A488 as a suitable FRET donor and A647N as the FRET acceptor. This reaction was performed sequentially as follows. In a first step, the protein was labeled by incubating the mutant with A488 dye (1:1 protein-to-A488 ratio) in 25 mM Tris buffer, pH = 8.5. The reaction mixture was allowed to shake at room temperature (21 °C) for 4 h. In the second step, we added A647N dye in a 1:3 protein-to-A647N ratio, to enhance the dye reactivity, leaving the reaction running overnight. The dye-maleimide solutions were prepared immediately before starting the labeling reactions. All the labeled constructs were purified through PD-10 desalting columns (PD-10 Sephadex G-25M, GE Healthcare). The efficiency of the labeling reactions was estimated, as specified by the provider, by recording the absorbance at 280, 501 and 650 nm using a NanoDrop 2000 UV-visible spectrophotometer (Thermo Fisher Scientific, Dreieich, Germany).

The singly labeled constructs (*Ce*PoFUT1-A488 and *Ce*PoFUT1-A647N) and the dual-labeled construct (*Ce*PoFUT1-A488-A647N) were stored at concentrations between 2.5–5.0 µM at 4 °C.

### 4.4. Inter-Dye Distance Estimation Using the Positioning and Screening Software (FPS)

Donor-acceptor distance estimation in single-molecule FRET experiments is usually overestimated, especially when the FRET efficiency is high, due to the flexibility of the dyes’ linkers and other sources of uncertainty. To have a better estimation of the three-dimensional space occupied by the donor and the acceptor dyes and, thus, of donor-acceptor distance, we employed the computational method developed by Claus Seidel and collaborators called FPS-screening and positioning software [37]. We used as input the crystal structure of *Ce*PoFUT1 (PDB 3ZY4) and selected as labeling positions the γ C of the H272 and M172 residues to attach the A488 maleimide and A647N maleimide dyes, respectively. The dimensions of the dyes were (i) A488: length = 18.8 nm, width = 4.5 nm, radius 1 = 5 nm, radius 2 = 4.5 nm, and radius 3 = 1.5 nm, as described in Vandenberk et al. [45]; and (ii) A647N: length = 21 nm, width = 4.5 nm, radius 1 = 7.15 nm, radius 2 = 4.5 nm, and radius 3 = 1.5 nm, as described in Kalinin et al. [37]. Using all these settings the space filled by the donor and the acceptor dyes is depicted in Figure 1e. The inter-dye distance distribution was obtained to be centered at 3.0 ± 1.1 nm. For this pair of dyes, we calculated a Förster distance, *R*_0_, value of 4.7 nm (considering free rotation of the fluorophores and a refractive index of the medium of 1.4). Once the donor-acceptor distance distribution is computed, the software allows to estimate the expected measured distance in a FRET experiment <*R*_DA_>*_E_* that was 3.5 nm, resulting in expected FRET efficiency of 0.856. These estimations are in excellent agreement with the experimental results.

### 4.5. Atomic Force Microscopy Imaging

Atomic force microscopy (AFM) allowed the direct visualization of single *Ce*PoFUT1 protein molecules to characterize the different conformational states upon ligand binding. The resolution achieved allowed to attribute single features to individual enzyme structures or their complexes. AFM measurements were performed in a MultiMode 8 AFM system (Bruker, Santa Barbara, CA, USA) using the tapping mode. V-shaped soft silicon nitride cantilevers with integrated pyramidal 2 nm ultrasharp tips exhibiting a spring constant of 0.06 N/m and a nominal resonant frequency of 18 KHz (SNL, Bruker Probes) were used.

AFM scanning requires the previous immobilization of the molecules on a nanoflat surface in order not to be dragged while scanning. 1 µM *Ce*PoFUT1 in 25 mM HEPES pH 7.0, 250 nM MgCl_2_ was incubated on freshly cleaved V-5 muscovite mica pieces (Electron Microscopy Sciences, Hatfield, PA, UK) for 10 min at room temperature. The best results were obtained immobilizing the samples on mica pretreated with 200 mM MgCl_2_ [46]. The pretreatment inverts the polarity of the net negative charge of mica sheets easing the immobilization through electrostatic absorption of enzyme molecules with also a negative charge at the working pH. Adsorption of enzymes on mica was previously evaluated and found they preserve the enzymatic activity, even later desorbed [47]. The enzyme was also incubated with ligands for 15 min under mild stirring at room temperature. GDP, GDP-Fuc, *Hs*EGF12 and *Hs*EGF11-12-13 were added at 10 µM. After sample incubation, the substrates were washed extensively to remove not adsorbed molecules and covered with the same buffer ready for measurements.

A series of clear AFM topography images were collected. AFM images were analyzed with the WSxM software [48]. At least, three samples per condition were assayed analyzing 10 images of 10 different areas of 500 nm^2^ [49]. Each feature was further analyzed using the zoom function of the WSxM program, performed without losing image information and discarding non-clear artifacts. The concentration of the enzyme incubated on the mica sheets was suitable to get isolated features (any individually identified element at the image) identified as molecules that could be analyzed individually, allowing the conformational analysis upon ligand binding.

In the case of incubation with *Hs*EGF11-12-13, samples were also incubated with 1 mM CaCl_2_ obtaining similar results that in the absence of the cation. The formation of complexes and subsequent measurements were also assayed in 25 mM MES pH 5.0 and 6.0 and the species observed were equivalent that the found when HEPES pH 7.0 was used. The observed species also do not depend on the presence or absence of magnesium. This cation, when present in the measurement buffer or used for substrate pretreatment, did not affect in the species found but rather the degree of immobilization thereof.

### 4.6. SMF-FRET Experiments

SMF-FRET experiments were performed in a MicroTime 200 time-resolved confocal fluorescence microscope (PicoQuant GmbH, Berlin, Germany) equipped with dual-color pulsed interleaved excitation (PIE) and multi-parameter fluorescence capabilities [50]. We employed two pulsed lasers at 470 and 635 nm (LDH-P-C-470 and LDH-P-635, from PicoQuant GmbH), alternated on a nanosecond time scale, to achieve PIE excitation and the detected fluorescence was separated by a 600DCXR dichroic mirror into two single-photon avalanche diode detectors (SPCM-AQR-14, Perkin-Elmer Optoelectronics, Hopkinton, MA, USA) after passing a 520/35 bandpass filter for the donor channel and a 685/70 bandpass filter for the acceptor channel.

For the SMF-FRET experiments, the dual-labeled *Ce*PoFUT1-A488-A647N construct was incubated during 30 min in a buffer containing 10 mM Tris, 1 mM EDTA, and 0.005% Tween 20, at pH 8.5, and with a 15-fold molar excess of the different substrates (*Hs*EGF11-12-13, *Hs*EGF11, GDP, GDP-Fuc). Then, the incubation mixture was diluted in several steps, reaching a final concentration of protein of 125 pM on the microscope slide.

For the dimerization experiments, the singly labeled constructs, *Ce*PoFUT1-A488 and *Ce*PoFUT1-A647N, were incubated in a 1:1 molar ratio at 1 µM during 30 min in the same working buffer described above. For the interaction with the substrates, they were added to this incubating mixture at a 15-fold molar excess. For SMF-FRET experiments, the mixture was diluted down to a final protein concentration of 40 pM.

For all the experiments, SMF traces were collected over 90 min and were repeated at least three times with different incubated samples. SMF traces were analyzed using SymPhoTime 32 software (PicoQuant GmbH). The fluorescence signals of the donor (*F*_A488_), FRET (*F*_FRET_) and directly excited acceptor (*F*_A647N_) were separated by selecting the corresponding detection channel and adequate time-gates to the overall traces. All the traces were corrected for the corresponding background signal, registering the buffer contribution in each channel; the spectral crosstalk of the donor dye in the acceptor detection channel; the fraction of direct acceptor excitation by the donor laser; and the γ factor that takes into account both the different detection efficiencies of the channels and the quantum yield of the dyes. All these correction factors were calculated as previously reported [34] using 45-basepair single-stranded DNA labeled with either A488 or A647N dyes and a dual-labeled (with both A488 and A647N dyes) 45-basepair double-stranded DNA as control samples. Single-molecule FRET bursts were accounted for using a 12 kHz threshold value for both the *F*_A488_ and the directly excited acceptor, *F*_A647N_, ensuring that a real FRET event contained both dyes. Furthermore, the fluorescence lifetime of the donor, *τ*_A488_, was estimated burstwise using the burst integrated fluorescence lifetime (BIFL) method [34,51]. Finally, home-coded scripts were used to obtain the ratio *F*_A488_/*F*_A647N_ of the coincident events, which is related to the stoichiometry of the interactions, and plot these data versus *τ*_A488_ in single-molecule correlograms.

For the quantification of the amount of dimers, the number of coincident bursts in the directly excited A488 and A647N channels, *F*_A488_ and *F*_A647N_ respectively, were selected and counted. Then, the association quotient, *Q*, corresponding to the fraction of coincident events in solution over all the detected single-molecule events [38] was obtained as:

(3) $$Q=\frac{n_{T}-n_{C}}{n_{A488}+n_{A647N}-(n_{T}-n_{C})}$$

where *n*_A488_ and *n*_A647N_ are, respectively, the burst rates in the *F*_A488_ and *F*_A647N_ channels, *n_T_* is the rate of coincident bursts and *n_C_* is the rate of chance coincidence events, accounting for random encounters of non-associated monomers [38].

## Data Availability

Data available upon request to the corresponding authors.
